# Loss of p16 and high Ki67 labeling index is associated with poor outcome in esophageal carcinoma

**DOI:** 10.18632/oncotarget.27507

**Published:** 2020-03-24

**Authors:** Frank Jacobsen, Jacob Kohsar, Florian Gebauer, Martina Kluth, Claudia Hube-Magg, Ronald Simon, Maximilian Bockhorn, Andrea Hinsch, Eike Burandt, Andreas M. Lübke, Stefan Steurer, Michael Tachezy, Guido Sauter, Jacob R. Izbicki, Wildemar Wilczak, Nathaniel Melling

**Affiliations:** ^1^Institute of Pathology, University Medical Center Hamburg-Eppendorf, Hamburg, Germany; ^2^Department of Surgery, University Hospital Cologne, Cologne, Germany; ^3^Department of General, Visceral and Thoracic Surgery, University Medical Center Hamburg-Eppendorf, Hamburg, Germany; ^*^These authors contributed equally to this work

**Keywords:** p16, 9p21, esophageal cancer, TMA, prognosis

## Abstract

The p16 tumor suppressor is coded by *CDKN2A* (9p21) and plays an important role during carcinogenesis and tumor progression in numerous tumor entities. The aim of our study was to evaluate the prognostic role of p16 expression and CDKN2A deletion in esophageal cancer (EC). Therefore, we analyzed p16 and KI67 expression by immunohistochemistry and 9p21 deletion by fluorescence *in-situ* hybridization on a tissue microarray including 398 adenocarcinomas (AC) and 293 squamous cell carcinomas (SCC) with clinical follow up-data. p16 positivity was found in 30.2% of AC and 13.9% of SCC and CDKN2A deletion in 32.1% of AC and 33.5% of SCC. In SCC p16 immunostaining correlated with low tumor stage (*P* = 0.014). In AC Ki67 positivity was associated with high tumor stage (*P* = 0.001), presence of lymph node metastasis (*P* = 0.009), high UICC stage (*P* = 0.001) and poor grading (*P* = 0.005). Overall survival (OS) was shorter for patients with high Ki67 labeling index (Ki67LI; *P* = 0.009) and negative p16 immunostaining (*P* = 0.026). In both histological tumor types, CDKN2A deletion showed no association with phenotype or outcome. Proportional cox-regression modeling revealed patients’ age, tumor stage, lymph node metastasis and Ki67 labeling index as independent prognostic markers in AC. In SCC, only patients’ age and tumor stage proved to be independent prognosticators. In summary, our study shows that loss of p16 expression and high Ki67LI is linked to shortened OS in AC. CDKN2A deletion shows no relevant association with tumor phenotype and patient outcome.

## INTRODUCTION

Despite recent advances in the management of the disease, esophageal cancer (EC) is the sixth most lethal malignant disease with nearly half a million novel cases and over 400,000 deaths worldwide [1]. Multimodal therapy including neoadjuvant chemo-radiotherapy followed by surgical resection of the cancer and regional lymph nodes represents the standard of care in this tumor entity [2]. Esophageal adenocarcinoma (AC) and squamous cell carcinoma (SCC) account for >90% of all malignant neoplasms of the esophagus. In recent years, the incidence for both tumor entities changed in Western countries with an increase of AC and a decrease of SCC, which is likely caused by changes in lifestyle habits [3]. Today, therapeutic decisions as to whether a patient receives neoadjuvant treatment, operation or palliative treatment are made according to the preoperative TNM staging [4]. However, esophageal cancer is highly heterogeneous and tumors with identical TNM stage demonstrate marked differences in clinical course and treatment response. Thus, the identification of markers predicting malignant potential and prognosis are of great importance.

The p16 tumor suppressor has been reported to play a pivotal role in cancer, since it inhibits cyclin-dependent kinases (CDKs) 4 and 6 at the G1 to S-phase transition of the cell cycle and thus prevents phosphorylation of the retinoblastoma (RB1) protein [5]. Maintaining hypophosphorylation of RB family members promotes binding to E2F1 and leads to G1 cell cycle arrest [6]. p16 is encoded by the *CDKN2A* gene localized on chromosome 9p21 within the INK4/ARF locus (reviewed in [7]). p16 plays an important role during carcinogenesis and tumor progression in numerous tumor entities including cancers of the colon, liver, gall bladder, and skin (reviewed in [8]). Its expression is associated with unfavorable or favorable tumor phenotype depending on the analyzed tumor entity (reviewed in [6, 8]). In EC, different types of p16 inactivation have been described, such as homozygous and heterozygous *CDKN2A* deletions, deleterious point mutations and p16 promoter methylation [9–13]. Previous studies additionally suggest, that alterations of p16 occur early during tumorigenesis as they are commonly seen in Barret’s dysplasia and peritumoral mucosa [14].

To elucidate the potential role of both p16 expression and CDKN2A deletion as prognostic biomarkers we examined our preexisting EC tissue microarray (TMA) built from tumor samples of more than 690 individual EC patients. The database attached to this TMA contains comprehensive molecular, pathological and clinical follow up data.

## RESULTS

### p16 and Ki67 immunohistochemistry (IHC)

**Figure 1:**
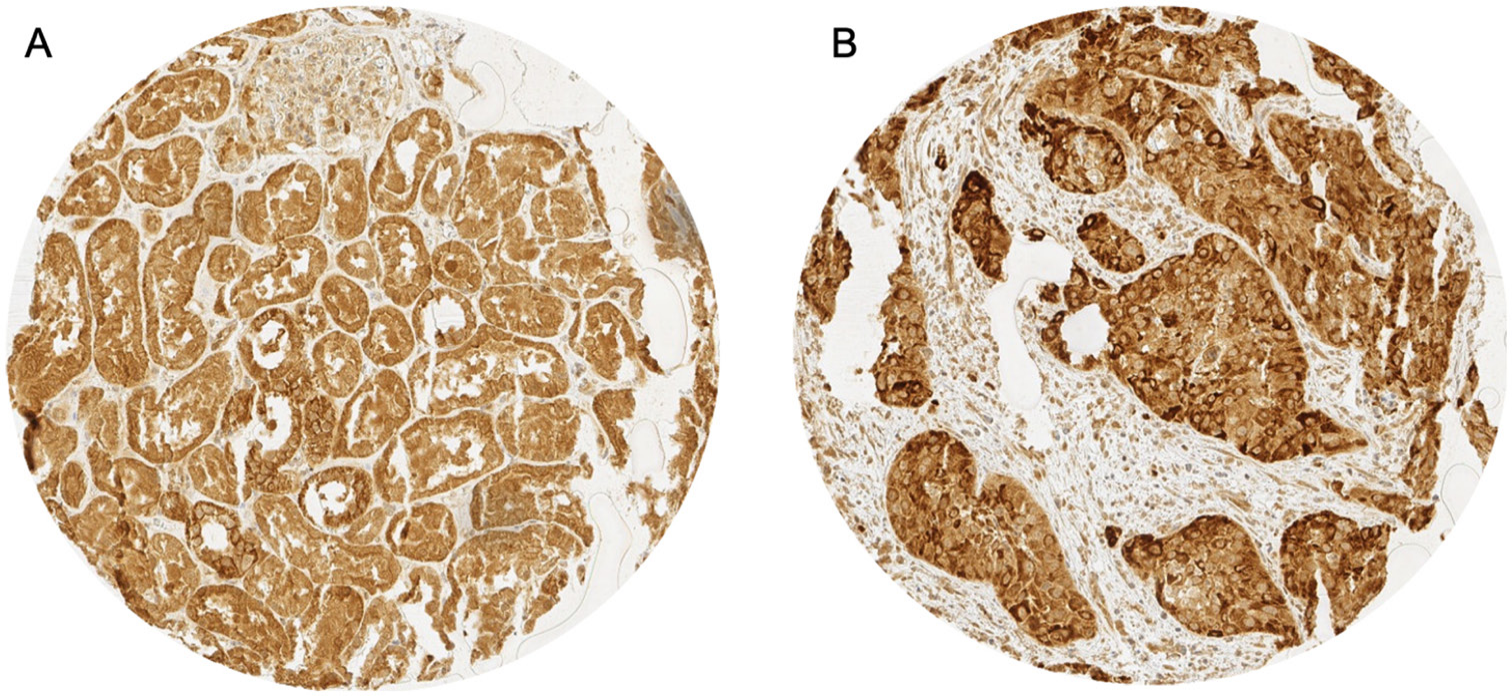
Representative images of p16 immunostaining in (**A**) p16 cytoplasmatic (red arrow) and nuclear (blue arrow) staining in adenocarcinoma and (**B**) p16 cytoplasmatic staining in squamous cell carcinoma.

p16 immunostaining was interpretable in 351 AC and 280 SCC. Non-informative cases were due to lack of tissue samples or absence of unequivocal cancer tissue in the TMA spot. 30.2% (*N* = 106) of all AC and 13.9% (*N* = 39) of SCC showed positive staining for p16. Representative images of p16 immunostaining in AC and SCC are given in Figure 1. p16 positivity was not associated with any clinical parameters in AC (Table 1) whereas in SCC positive p16 immunostaining correlated with gender (*P* = 0.032) and low tumor stage (*P* = 0.014, Table 2).

**Table 1:** Association of p16 immunostaining, *CDKN2A* FISH and Ki67LI with clinico-pathological parameters in adenocarcinoma

		p16 IHC				*CDKN2A* FISH					Ki67 IHC				
		*n*	neg.	pos.	*p*	*n*	no del	het del	hom del	*p*	*n*	<10%	10–80%	>80%	*p*
All tumors		351	69.8	30.2		202	67.9	25.7	6.4		312	54.5	40.4	5.1	
Age group	<65 yrs	120	69.2	30.8	0.852	63	58.8	31.7	9.5	0.153	105	54.3	41.0	4.8	0.973
	>65 yrs	231	70.1	29.9		139	72.0	23.0	5.0		207	54.6	40.1	5.3	
Sex	male	293	71.0	29.0	0.316	170	68.8	25.3	5.9	0.623	261	54.8	39.8	5.4	0.198
	female	56	64.3	35.7		31	61.3	29.0	9.7		50	54.0	44.0	2.0	
Tumor stage	pT1	75	66.7	33.3	0.426	30	80.0	16.7	3.3	0.281	62	77.4	22.6	0.0	0.001
	pT2	37	62.2	37.8		20	55.0	30.0	15.0		33	48.5	51.5	0.0	
	pT3	213	71.8	28.2		133	67.6	25.6	6.8		192	47.4	44.8	7.8	
	pT4	24	79.2	20.8		18	61.1	38.9	0.0		23	56.5	39.1	4.3	
Lymph node metastasis	pN0	115	69.6	30.4	0.298	56	66.0	28.6	5.4	0.734	95	66.3	30.5	3.2	0.009
	pN1	58	63.8	36.2		30	76.7	13.3	10.0		53	60.4	37.7	1.9	
	pN2	87	67.8	32.2		51	68.6	25.5	5.9		78	52.6	39.7	7.7	
	pN3	89	77.5	22.5		64	35.9	29.7	6.2		84	38.1	54.8	7.1	
UICC Stage	I	77	68.8	31.2	0.887	34	73.6	17.6	8.8	0.535	62	79.0	21.0	0.0	0.001
	II	46	67.4	32.6		23	60.9	39.1	0.0		39	43.6	51.3	5.1	
	III	188	72.3	27.7		118	67.0	25.4	7.6		173	50.3	43.9	5.8	
	IV	36	69.4	30.6		24	66.6	29.2	4.2		34	41.2	47.1	11.8	
Distant metastasis	M0	312	69.6	30.4	0.774	176	68.8	25.0	6.2	0.763	276	56.5	39.1	4.3	0.061
	M1	39	71.8	28.2		26	61.5	30.8	7.7		36	38.9	50.0	11.1	
Surgical resection margin	R0	259	68.0	32.0	0.075	144	68.0	26.4	5.6	0.737	223	57.8	38.1	4.0	0.063
	R1	83	78.3	21.7		52	65.4	25.0	9.6		81	45.7	46.9	7.4	
	R2	3	33.3	66.7		2	100.0	0.0	0.0		3	33.3	33.3	33.3	
Grading	G1	20	65.0	35.0	0.491	5	80.0	20.0	0.0	0.261	17	94.1	5.9	0.0	0.005
	G2	128	65.6	34.4		67	55.3	34.3	10.4		113	61.1	35.4	3.5	
	G3	191	73.3	26.7		126	73.0	22.2	4.8		173	45.7	48.0	6.4	
	G4	6	66.7	33.3		1	100.0	0.0	0.0		4	50.0	50.0	0.0	

**Table 2:** Association of p16 immunostaining, *CDKN2A* FISH and Ki67LI with clinico-pathological parameters in squamous cell carcinomas

		P16 IHC				*CDKN2A* FISH					Ki67 IHC				
All tumors		*n*	negative	positive	*p*	*n*	no del	het del	hom del	*p*	*n*	<10%	10–80%	>80%	*P*
		280	86.1	13.9		161	66.5	30.4	3.1		261	42.1	51.7	6.2	
Age group	<65 yrs	108	86.1	13.9	0.988	64	76.6	23.4	0.0	0.033	101	43.6	52.5	4.0	0.506
	>65 yrs	172	86.0	14.0		96	59.4	35.4	5.2		160	41.2	51.2	7.5	
Sex	male	206	88.8	11.2	0.032	118	65.2	31.4	3.4	0.884	191	41.4	53.9	4.7	0.211
	female	74	78.4	21.6		42	69.0	28.6	2.4		70	44.3	45.7	10.0	
Tumor stage	pT1	52	73.1	26.9	0.014	27	77.8	22.2	0.0	0.019	43	51.2	44.2	4.7	0.611
	pT2	56	83.9	16.1		34	64.7	23.5	11.8		54	42.6	53.7	3.7	
	pT3	153	90.8	9.2		86	64.0	36.0	0.0		147	39.5	52.4	8.2	
	pT4	18	88.9	11.1		13	61.5	30.8	7.7		17	41.2	58.8	0.0	
Lymph node metastasis	pN0	137	83.9	16.1	0.407	77	64.9	31.2	3.9	0.421	126	43.7	50.8	5.6	0.897
	pN1	60	86.7	13.3		32	78.1	21.9	0.0		58	34.5	56.9	8.6	
	pN2	55	85.5	14.5		35	54.3	40.0	5.7		51	45.1	49.0	5.9	
	pN3	27	96.3	3.7		16	75.0	25.0	0.0		25	44.0	52.0	4.0	
UICC stage	I	69	76.8	23.2	0.059	41	70.7	22.0	7.3	0.493	60	46.7	50.0	3.3	0.587
	II	65	87.7	12.3		32	62.5	37.5	0.0		63	42.9	49.2	7.9	
	III	95	91.6	8.4		56	66.1	32.1	1.8		90	40.0	51.1	8.9	
	IV	49	85.7	14.3		31	64.5	32.3	3.2		47	38.3	59.6	2.1	
Distant metastasis	M0	230	86.5	13.5	0.654	127	67.0	29.9	3.1	0.931	213	43.2	49.8	7.0	0.255
	M1	50	84.0	16.0		33	63.7	33.3	3.0		48	37.5	60.4	2.1	
Surgical resection margin	R0	209	84.7	15.3	0.284	118	67.8	28.8	3.4	0.525	195	43.1	50.3	6.7	0.228
	R1	56	87.5	12.5		32	59.4	40.6	0.0		50	32.0	62.0	6.0	
	R2	13	100.0	0.0		9	77.8	22.2	0.0		14	64.3	35.7	0.0	
Grading	G1	5	100.0	0.0	0.615	1	0.0	100.0	0.0	0.651	3	0.0	100.0	0.0	0.002
	G2	176	86.4	13.6		106	67.0	30.2	2.8		164	48.2	49.4	2.4	
	G3	98	84.7	15.3		52	67.4	28.8	3.8		93	33.3	53.8	12.9	

Ki67LI was evaluable in 312 AC and 261 SCC. Immunostaining was low in 54.5% (*N* = 170) of AC, moderate Ki67LI was seen in 40.4% (*N* = 126) and strong KI67LI in 5.1% (*N* = 16). In SCC, low Ki67LI was present in 42.1% (*N* = 110), moderate in 51.7% (*N* = 135) and strong in 6.2% (*N* = 16). Association with clinical data was found in AC between high-level Ki67 staining and high tumor stage (*P* = 0.001), presence of lymph node metastasis (*P* = 0.009), high UICC stage (*P* = 0.001) and poor grading (*P* = 0.005, Table 1). For SCC, merely a link between Ki67 immunostaining and poor grading (*P* = 0.002, Table 2) was revealed.

### CDKN2A fluorescence *in-situ* hybridization (FISH)

**Figure 2:**
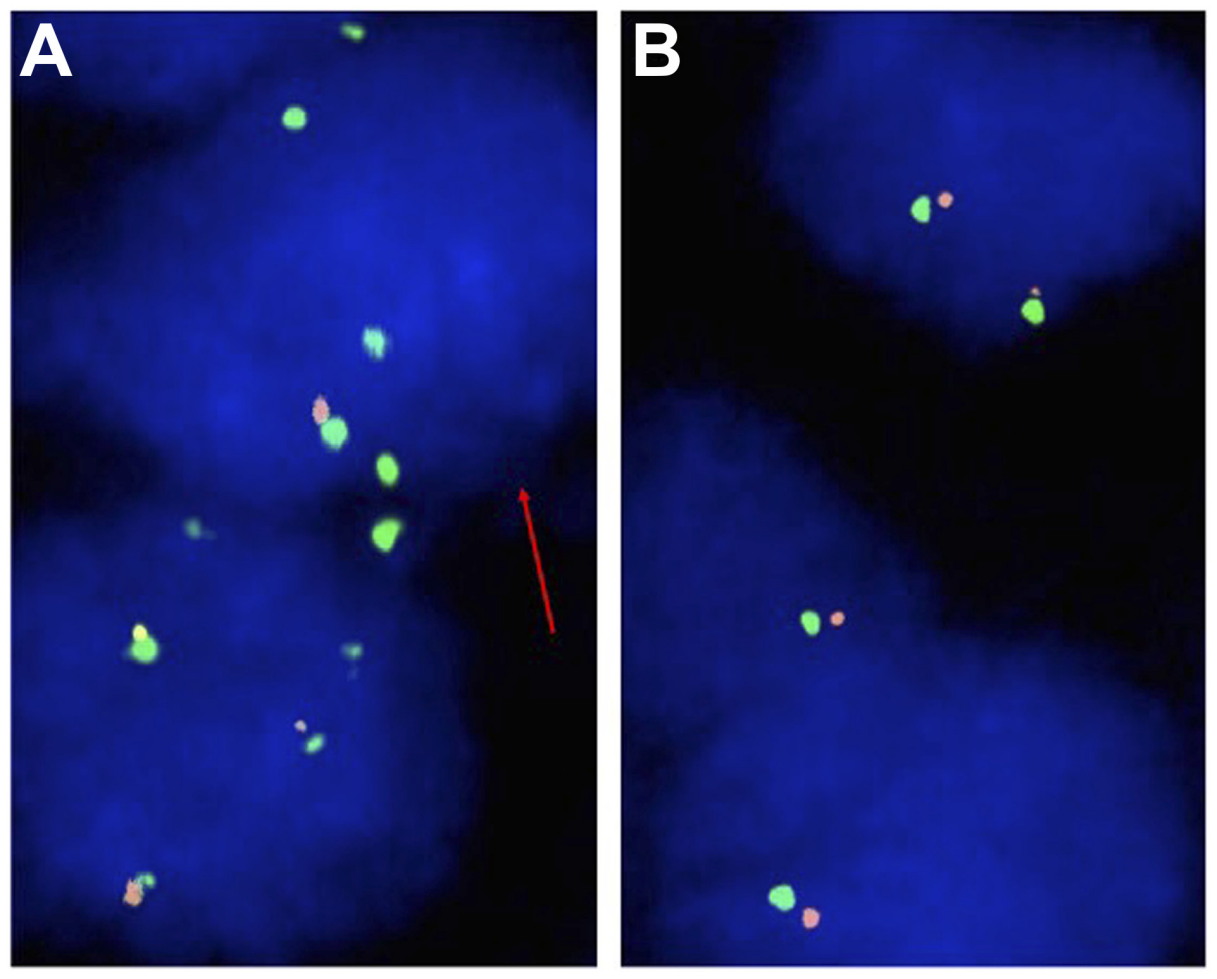
Representative FISH images of CDKN2A (**A**) Heterozygous CDKN2A deletion indicated by the lack of one orange CDKN2A signal and two green centromere 9 signals in the tumor cell nucleus (red arrow) and (**B**) Normal CDKN2A copy number indicated by two orange CDKN2A signals and two green centromere 9 signals.

*CDKN2A* FISH analysis was interpretable in 202 (50.8%) samples of AC and 161 (54.8%) samples of SCC. Non-informative cases were caused by inefficient hybridization, missing tissue spots or absence of representative tumor tissue on the TMA spot. Representative images are shown in Figure 2. Homozygous *CDKN2A* deletions were detectable in 13 samples (6.4%) and heterozygous deletions in 52 samples (25.7%) of AC. In SCC, homozygous deletions were detectable in 5 patients (3.1%) and heterozygous deletions in 49 tissue spots (30.4%).

In AC no links were evident between deletion rates and clinico-pathological parameters (Table 1), while in SCC *CDKN2A* deletions were associated with patients’ age (*P* = 0.033) and tumor stage (*P* = 0.024, Table 2).

A correlation between p16 immunostaining and *CDKN2A* deletion was found for AC (*P* = 0.039) but not for SCC (*P* = 0.610). However, in both histological tumor types, all cases with homozygous gene deletion were negative for p16 immunostaining (data not shown).

### Combination of p16 and Ki67 IHC with CDKN2A deletions

Since a correlation between p16 immunostaining and *CDKN2A* deletion was found for AC a combined analysis of IHC and FISH was performed.

Data on both p16 immunostaining and *CDKN2A* deletion were available from 172 AC and 142 SCC. In AC, 37 samples (21.5%) were immunopositive for p16 and showed no *CDKN2A* deletion, while in SCC, combined p16 positivity and absence of *CDKN2A* deletion was seen in 16 samples (9.2%). There was no correlation detectable between clinical parameters and the combination of p16 expression with *CDKN2A* deletion.

Furthermore, no association was found between p16 and Ki67 immunostaining for either histological type (AC: *P* = 0.400 and SCC: *P* = 0.764). In addition, links between Ki67 immunostaining and *CDKN2A* deletion status were also not detectable (AC: *P* = 0.172; SCC: *P* = 0.712).

### Survival analysis

**Figure 3:**
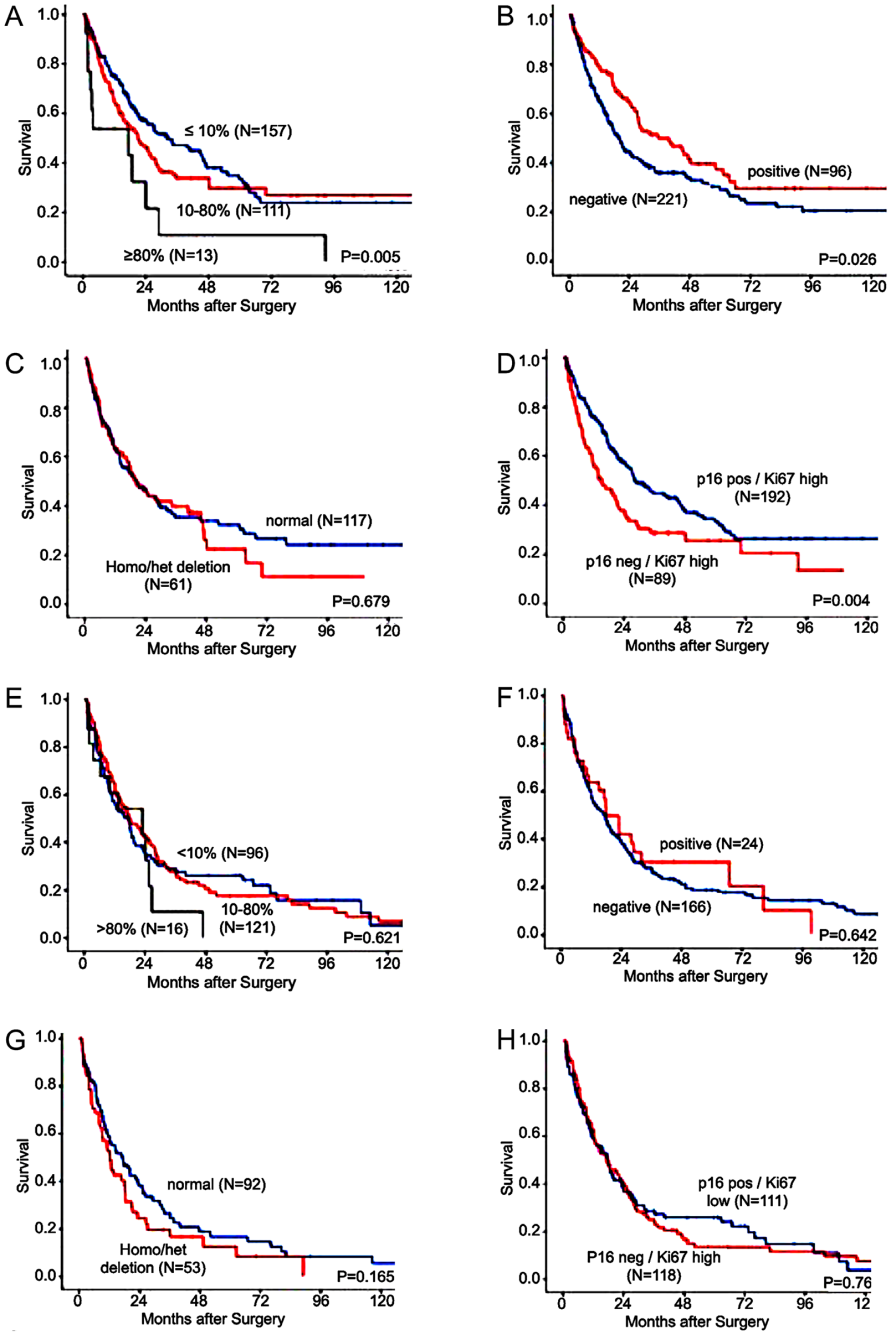
Association of immunohistochemistry (IHC) and fluorescence *in-situ* hybridization (FISH) results with the median overall-survival in patients with esophageal cancer. (**A**) KI67 immunostaining divided into ≤10%, 10–80%, and ≥80% Ki67 stained tumor cells and overall survival in adenocarcinoma, (**B**) p16 immunostaining divided in negative and positive and overall survival in adenocarcinoma, (**C**) CDKN2A FISH analysis divided in deletion (homozygous and heterozygous) and overall survival in adenocarcinoma, (**D**) combined p16 staining and Ki67LI and overall survival in adenocarcinoma, (**E**): KI67 immunostaining divided into ≤10%, 10–80%, and ≥80% Ki67 stained tumor cells and overall survival in squamous cell carcinoma, (**F**) p16 immunostaining divided in negative and positive and overall survival in squamous cell carcinoma (**G**): CDKN2A FISH analysis divided in deletion (homozygous and heterozygous) and overall survival in squamous cell carcinoma, (**H**): combined p16 staining and Ki67LI and overall survival in squamous cell carcinoma.

Kaplan-Meier survival analysis for OS in AC showed shortened survival rates for patients with high Ki67 labeling index (*P* = 0.009, Figure 3A). Negative p16 immunostaining was also associated with a shortened overall survival (OS) compared to cancers showing p16 staining (*P* = 0.026, Figure 3B). *CDKN2A* deletions had no influence on the OS in AC (*P* = 0.679, Figure 3C).

Combined analysis of Ki67 and p16 IHC suggested a superior prognostic value as compared to analysis of Ki67 and p16 expression alone, revealing favorable prognosis for patients with a combination of positive p16 immunostaining and low Ki67LI (*P* = 0.004, Figure 3D).

For SCC, neither the Ki67 labeling index (*P* = 0.621, Figure 3E) nor the p16 immunostatus (*P* = 0.682, Figure 3F) nor the *CDKN2A* deletion status (*P* = 0.165, Figure 3G) nor the combined analysis of Ki67LI and p16 (*P* = 0.7610, Figure 3H) reached statistical significance as a prognostic marker.

### Multivariate Cox-regression analysis

Multivariate Analyses were performed evaluation the prognostic relevance of Ki67LI, p16 expression and CDKN2A deletion in relationship to patients’ age, sex and clinic-pathological parameters (pT, pN, pM, UICC stage, Grade, resection margin) in AC and SCC. For AC, the proportional cox-regression model revealed higher patients’ age (*P* = 0.0050), lymph node metastasis (*P* = 0.0070), resection margin (*P* = 0.0010) and the Ki67LI (*P* = 0.0040) as independent prognostic markers. In SCC, only tumor stage (*P* = 0.0260) proved to be independent prognosticators (Table 3).

**Table 3:** Multivariate Cox-regression model for esophageal adenocarcinoma and squamous cell carcinoma

	Hazard ratio	Adenocarcinoma 95% confidence interval		*p* value	Hazard Ratio	Squamous cell carcinoma 95% confidence interval		*p* value
		lower	upper			lower	upper	
Age group	2.008	1.235	3.264	0.005	1.104	0.733	1.664	0.635
Sex (male vs. female)	0.675	0.371	1.225	0.196	0.871	0.547	1.384	0.558
Tumor stage (pT)	1.410	0.992	2.004	0.055	1.504	1.050	2.156	0.026
Lymph node metastsis (pN)	1.501	1.119	2.013	0.007	1.038	0.776	1.388	0.803
UICC stage	1.030	0.586	1.813	0.918	1.017	0.648	1.595	0.943
Distant metastasis (M)	0.961	0.426	2.169	0.924	1.377	0.632	2.998	0.421
Resection margin (R)	2.161	1.385	3.373	0.001	1.097	0.757	1.590	0.626
Grading (G)	0.920	0.587	1.440	0.715	1.163	0.755	1.792	0.494
Ki67 labeling index	1.714	1.183	2.481	0.004	0.871	0.604	1.257	0.462
p16 immunostaining	1.078	0.680	1.708	0.750	0.954	0.541	1.682	0.869
CDKN2A deletion	1.118	0.826	1.514	0.469	1.184	0.813	1.722	0.378

## DISCUSSION

The results of our study show that loss of p16 expression – but not 9p21 deletion - and high Ki67LI are prognosticators of poor survival in AC of the esophagus.

Aim of the present study was to assess whether p16 alterations are associated with adverse clinical outcome in patients with EC. Therefore, in our analysis, 351 AC and 280 SCC were analyzed by IHC (p16 expression) and FISH (CDKN2A deletion). Under the selected experimental conditions, our immunohistochemical analysis revealed 30% of AC and 14% of SCC positive for p16. Immunohistochemical analysis of p16 expression in EC is available in two studies only with expression rates varying between 15–60% and also depending on the underlying histological subtype [9, 15]. Discrepancy in expression frequencies is rather common when protein detection is performed using IHC. This is likely caused by variable immunohistochemistry conditions. It is well known that varying antibody conditions lead to significant changes in the rate of positive cases [16]. This is all the more expected in the case of ubiquitously expressed proteins, such as p16. Therefore, in some cases it may be difficult to differentiate between truly p16 negative cancers and lacking sensitivity.

Deletion rates differed between AC and SCC from about 25% in AC to almost 50% heterozygous deletions in SCC. Our deletion rate in AC is somewhat lower than reported in other studies [13, 14, 17–19], which is partially caused by more stringent criteria for defining *CDKN2A* deletions. These were applied to avoid false deletion calling due to truncation of the nuclei during tissue sectioning. Loss of heterozygosity (LOH) studies revealed LOH frequencies in SCC ranging from 65–79% [17–19]. However, LOH assays are influenced by ploidy changes, which are frequent in EC. These inevitably impact the assay sensitivity and vary markedly between individual tumors. In contrast, FISH allows for precise gene copy number determination in individual cells, rendering it independent of cancer tissue purity or aneuploidy. FISH is, thus, considered the gold standard for gene copy number analysis.

p16 is known to be a major tumor suppressor protein and its alteration has been associated with tumor progression in different entities [8]. p16 loss was linked to shortened overall survival and positive nodal stage in AC in our study. Other parameters, such as tumor stage and grade barely failed to reach statistical significance, which was probably first of all due to the low number of samples in some subgroups. p16 loss or downregulation but also its clear overexpression has been evaluated as negative prognosticators in several tumor types. Almost 50% of all tumors show p16 inactivation as one of the main drivers during carcinogenesis including pancreatic and biliary, head and neck, lung, bladder and colon carcinoma [20, 21]. Different mechanisms of p16 inactivation have been described earlier including promoter hypermethylation, point and missense mutations, loss of heterozygosity (LOH) and genetic deletions [13].

As p16 is considered a tumor suppressor and negative regulator for cell proliferation we correlated p16 expression with the cell proliferation marker Ki67 to analyze whether the loss of p16 was associated with increased cell proliferation. Ki67 is a known index marker for aggressive tumor behavior, including dedifferentiation. In line with the results of our analysis, high Ki67 indices have been linked to advanced tumor stages in EC [22, 23]. That Ki67 had independent prognostic value in our multivariate analysis, but not p16, suggests that Ki67 may be more promising candidate for clinical testing than p16. Interestingly, an association between loss of p16 and high Ki67LI was not seen, therefore regulative functions mediated by p16 protein expression are more complex than solely explained by increased cell proliferation. Although, possibly, the high rate of cancers with low Ki67LI experienced in our study may be misleading in this context rendering some results insignificant. Thus, the link may be masked by a slightly less sensitive Ki67 staining protocol than in the above mentioned study by Takeuchi [22, 23].

Another aim was to assess the relationship between *CDKN2A* deletions and p16 expression. A correlation was found for AC but not for SCC, which may be explained by different molecular mechanisms for inactivation of *CDKN2A* in the two histological tumor types. Inactivation of *CDKN2A* involves four types of genetic alterations: homozygous deletion, promoter hypermethylation, loss of heterozygosity and point mutation. Homozygous deletion and promoter hypermethylation constitute the majority of p16 alterations, but some cancers are known to prefer specific types of alterations. Promoter hypermethylation has been described as a main pathway for inactivation of *CDKN2A* for SCC of the esophagus [6, 24], while deletion appears to happen early in the development of Barrett’s mucosa [13], a recognized precursor lesion for esophageal AC.

Deletions are a common mechanism of gene inactivation for numerous tumor suppressor genes. As expected, all cancers with bi-allelic (homozygous) CDKN2A deletion completely lacked p16 expression, which indirectly validates our experimental approaches both for FISH and IHC. Differences in p16 expression levels in samples with or without heterozygous CDKN2A deletion demonstrate that EC cells have the ability to compensate the loss of one p16 (*CDKN2A*) allele, either by increased transcriptional activation of the remaining allele or by increased stabilization of p16 protein or mRNA (reviewed in [6, 24]). This also explained the absent of significant associations between CDKN2A deletions and clinic-pathological parameters as well as overall survival in our study. However, rare (5–6% depending on the histological subtype) biallelic *CDKN2A* deletions lead to catastrophic events for the cell with total loss of p16 expression.

It is a limitation of our study that the numbers of samples interpretable for p16 and Ki67 or p16 and CDKN2A are different and that combined analyses of these markers could only be made in subsets of the cancers. However, we do not consider this as a serious issue given that the total numbers of patients in these subsets are still comparatively high.

In summary, the results of our study show that loss of p16 expression - but not CDKN2A deletion - is linked to shortened overall survival in patients with esophageal AC. Furthermore, strong Ki67LI is an independent prognosticator of poor survival in AC. Rare homozygous 9p21 deletions can be considered as catastrophic events leading to complete loss of p16 expression.

## MATERIALS AND METHODS

### Patients

For this study, specimens from patients that had undergone tumor resection in curative intent between 1992 and 2014 at the University Medical Center, Hamburg-Eppendorf were included. Tissue samples from 691 patients were analyzed including 398 AC and 293 SCC. All data including sex, tumor histology, size, lymph node metastasis and disease stage (UICC 7th edition) were obtained by reviewing a combination of clinical and pathological records, outpatient clinic medical records, epidemiological cancer surveillance data bases and by communication with the patients and their attending physicians. Overall (raw) survival was used as the clinical endpoint in this study. Clinical follow-up data were available for 635 patients with a median follow-up of 13.4 months (1 to 208.3 months). All resections were performed as en-bloc esophagectomies with radical two field lymph node dissection. Fifty patients underwent neoadjuvant therapy (AC *n* = 30, SCC *n* = 20) but therapy was unknown for the remaining patients. Patients who died within 30 days due to postoperative complications were not considered for survival analysis. The study was approved of by the Ethics Committee of the Chamber of Physicians of Hamburg, Germany (WF-035/14).

### TMA construction

The TMA was constructed as previously described [25]. In brief, tissue cores were obtained from formalin-fixed paraffin-embedded (FFPE) tissue blocks from patients with pathologically proven EC. Representative areas of the tumor were selected based on hematoxylin–eosin staining. 691 tissue cylinders with a diameter of 0.6 mm were punched from the ‘‘donor’’ tissue blocks using a custom-made semi-automatic robotic precision instrument and placed into one empty recipient paraffin block. The resulting TMA blocks were used to produce 4 µm sections that were transferred to an adhesive-coated slide system (Instrumedics Inc., Hackensack, NJ).

### IHC

Freshly cut TMA sections were immunostained on one day and in one experiment. Slides were deparaffinized, rehydrated, washed in DAKO buffer (K8002) and transferred to a DAKO Link 48 autostainer device. The immunohistochemical staining of p16 was performed with the commercially available CINtec p16 Histology Kit (Dilution 1:150, Cat# 725-4713, Ventana Medical Systems Inc., Arizona, USA) according to the manufacturer’s instructions. Staining was evaluated according to the following scoring system: The staining intensity (0, 1+, 2+, and 3+) and the fraction of positive tumor cells were recorded for each tissue spot. A final score was built from these 2 parameters according to the following score as previously described [26]: Negative stainings showed complete absence of staining. Weak scores had staining intensities of 1+ in ≤70% of tumor cells or of 2+ in ≤30% of tumor cells. Moderate scores had staining intensities of 1+ in >70% of tumor cells, staining intensities of 2+ in >30% but in >70% of tumor cells or staining intensities of 3+ in ≤30% of tumor cells. Strong scores had staining intensities of 2+ in ≤70% of tumor cells or staining intensities of 3+ in ≤30% of tumor cells. For statistical analysis all cancers with weak, moderate and strong staining was grouped as p16 positive. For Ki67 immunostaining standard indirect immunoperoxidase procedures were used for the detection of Ki67 (abcam, clone SPM171, dilution 1:150). Sections were heated in an autoclave at 121°C for 10 minutes in citrate puffer (pH 9.0). Diaminobenzidine was used as a chromogen, and sections were counterstained with Mayer’s haematoxylin. Ki67 staining was evaluated as follows: the number of invasive cancer cell nuclei that were positive for Ki67 immunostaining was divided by the total number of invasive cancer cell nuclei present in a histological sample resulting in the Ki67 labeling index (Ki67LI). For each spot, the procedure was repeated three times and the mean value was calculated. Three groups were stratified with scores ranging from 0 to 2 (low, moderate, strong). 0 represented tissue spots with Ki67LI <10%, 1 stood for Ki 67LI between 10 and 80% and 2 for Ki67LI >80%.

### FISH

Four micrometer TMA sections were also used for FISH. For proteolytic slide pretreatment, a commercial kit was used (paraffin pretreatment reagent kit; Abbott, Wiesbaden, Germany). TMA sections were deparaffinized, air-dried, and dehydrated in 70%, 85%, and 100% ethanol, followed by denaturation for 5 min at 74°C in 70% formamide 2× SSC solution. The commercial Vysis CDKN2A/CEP 9 FISH probe kit (#04N61-020; Abbott, Wiesbaden, Germany) was used for detection of the 9p21 status. Hybridization was performed overnight at 37°C in a humidified chamber. Slides were subsequently washed and counterstained with 0.2 µmol/L 4′-6-diamidino-2-phenylindole in antifade solution. Stained slides were manually interpreted with an epifluorescence microscope, and the predominant FISH signal numbers were recorded in each tissue spot. Presence of fewer CDKN2A signals than centromere 9 probe signals in at least 60% of the tumor nuclei was considered to inidicate heterozygous deletion. Complete absence of CDKN2A signals in the tumor cells, but presence of centromere 9 and CDKN2A signals in adjacent normal cells, was considered to be a homozygous deletion. Tissue spots lacking any detectable CDKN2A signals in all (tumor and normal cells) or lack of any normal cells as an internal control for successful hybridization of the CDKN2A probe were excluded from analysis. These thresholds are based on our previous study analyzing PTEN deletions on a prostate cancer TMA where our approach resulted in a 100% concordance with aCGH data [27].

### Statistical analysis

SPSS Statistics for Mac (Version 17, SPSS) was used for statistical analysis. Contingency tables and the chi^2^-test were performed to search for associations between molecular parameters and tumor phenotype. Survival curves were calculated according to Kaplan-Meier. The Log-Rank test was applied to detect significant differences between groups. Cox proportional hazards regression analysis was performed to test the statistical independence and significance between pathological, molecular and clinical variables. Separate analyses were performed using different sets of parameters available either before or after prostatectomy. All tests were two-sided. *P* values <0.05 were considered statistically significant.

## Abbreviations

AC: adenocarcinomas
EC: esophageal cancer
FFPE: formalin-fixed paraffin-embedded
FISH: fluorescence *in-situ* hybridization
IHC: immunohistochemistry
Ki67LI: Ki67 labeling index
OS: overall survival
SSC: squamous cell carcinomas
TMA: tissue microarray
